# Dopaminergic changes in the subgenual cingulate cortex in dementia with lewy bodies associates with presence of depression

**DOI:** 10.1038/s41398-025-03298-3

**Published:** 2025-03-20

**Authors:** Lina Gliaudelytė, Steven P. Rushton, Rolando Berlinguer-Palmini, Alan J. Thomas, Christopher M. Morris

**Affiliations:** 1 Alzheimer’s Society Doctoral Training Centre, Edwardson Building, Newcastle University, Newcastle upon Tyne, UK; 2 School of Natural and Environmental Sciences, Agriculture Building, Newcastle University, Newcastle upon Tyne, UK; 3 Bioimaging Unit, Leech Building, Newcastle University, Framlington Place, Newcastle upon Tyne, UK; 4 Biomedical Research Building, Newcastle University, Newcastle upon Tyne, UK; 5 Queen Elizabeth Hospital, Queen Elizabeth Avenue, Gateshead, Tyne and Wear UK; 6 Newcastle Brain Tissue Resource, Edwardson Building, Newcastle University, Newcastle upon Tyne, UK

**Keywords:** Molecular neuroscience, Neuroscience

## Abstract

In addition to the core clinical features of fluctuating cognition, visual hallucinations, and parkinsonism, individuals with dementia with Lewy bodies (DLB) frequently experience chronic and debilitating major depression. Treatment of depression in DLB is hampered by a lack of available effective therapies and standard serotonergic medication for major depressive disorder (MDD) is typically ineffective. Dysfunction of dopaminergic neurotransmission contributing to anhedonia and loss of motivation has been described in MDD. The subgenual anterior cingulate cortex (sgACC) is important in mood regulation and in the symptomatic expression of depression, displaying structural, functional and metabolic abnormalities in MDD. To assess dopaminergic and serotonergic synaptic changes in DLB, post mortem sgACC tissue from DLB donors with and without depression was investigated using high-resolution stimulated emission depletion (STED) microscopy, as well as Western and dot blotting techniques. STED imaging demonstrated the presence of α-synuclein within individual dopaminergic terminals in the sgACC, α-synuclein presence showing a significant positive correlation with increased synaptosomal associated protein 25 kDa (SNAP25) volumes in depressed DLB cases. A reduction in dopaminergic innervation in the sgACC was observed in DLB cases with depression compared to controls (*p* < 0.001), but not in non-depressed DLB donors, along with reduced levels of multiple dopaminergic markers and receptors. Limited alterations were observed in serotonergic markers. Our work demonstrates a role for dopaminergic neurotransmission in the aetiology of depression in DLB. Careful and selective targeting of dopaminergic systems in the sgACC may be a therapeutic option for treatment of depression in DLB.

## Introduction

Dementia with Lewy bodies (DLB) is a significant cause of morbidity in older populations, representing between 5–20% of all clinical dementia cases [1, 2]. DLB shows core symptoms of fluctuating cognition, parkinsonism, the presence of recurrent complex visual hallucinations and REM sleep behaviour disorder [3]. Psychiatric features are highly prevalent in many DLB patients, with major depression present in 50–80% of cases [4]. Depression is frequently present at prodromal DLB stages, persisting throughout the clinical course [5]. Depression in dementia is associated with poor quality of life, increased morbidity and more rapid cognitive decline, and treatment would have significant patient benefit [6, 7].

The subgenual anterior cingulate cortex (sgACC) is a key brain region associated with mood and anxiety, and relays emotional information between limbic, cortical and subcortical regions [8, 9]. Abnormalities in sgACC activity, connectivity and grey matter volume have been shown in depressive disorders [10], with sgACC used as a target for treating treatment resistant depression with deep brain stimulation [11, 12].

The monoamine hypothesis of depression in unipolar major depressive disorder (MDD), suggests that reduction in serotonergic and noradrenergic neurotransmission underpins depressive symptoms [13]. Considerable evidence supports the monoamine hypothesis in individuals with MDD, with reduced 5HT concentrations observed in serum and reduced 5HIAA in CSF in patients, along with decreased serotonin 5HT1_A_ and 5HT2_A_ binding in anterior cingulate cortex (ACC) in MDD [14, 15]. However, reduced levels of 5HT as a basis of depression have been challenged by some, primarily based on reduced and delayed response to 5HT based therapies [16, 17]. Data on serotonergic treatment of depression in DLB is lacking, and in depression in Parkinson’s disease (PD) serotonergic treatment shows minimal effects in a few small-scale trials [18, 19].

Dopamine plays a role in reward and stress [20] with dysfunction of dopaminergic neurotransmission within the mesolimbic and mesocortical systems contributing to anhedonia and loss of motivation in depression [21]. A significant reduction in dopamine transporter (DAT) uptake is observed in anhedonic depressed patients [22], along with increased striatal D2 receptor binding in MDD suggesting decreased dopamine turnover [23]. Depression in PD is associated with nigral and mesolimbic dopaminergic pathway dysfunction with reduced ventral tegmental area (VTA), cingulate and amygdala volumes on MRI, and reduced [^11^C]RTI-32 DAT uptake in limbic regions in PD patients, with depression correlated with loss of dopamine projections from the VTA [24] and substantia nigra (SN) [25]. Whilst PD with dementia shows reduced VTA neurone numbers, altered VTA neurone number in DLB is mild although neurone dysfunction has not been established [26]. However, both DLB and PD show SN dopaminergic neurone loss and the contribution of SN dopamine depletion is unknown [26]. What role dopaminergic system plays in DLB patients with depression is unclear.

In this study, we investigated dopaminergic parameters in relation to depression in DLB. We focussed on the subgenual anterior cingulate cortex (sgACC) due to its important role in mood regulation and the symptomatic expression of depression, and as a region vulnerable to α-synuclein pathology in DLB [10, 27]. Little is known about how changes in dopaminergic neurotransmission in the sgACC might relate to development of depression in DLB, despite high levels of α-synuclein and other neurodegenerative pathology within the sgACC [28]. We therefore assessed dopaminergic innervation in the sgACC using a combination of stereology, high resolution stimulated emission depletion (STED) microscopy, and protein determination using post-mortem tissue samples from DLB cases with and without depression to investigate associations with depression.

## Materials and methods

### Ethics approval

Ethical approval met all UK national regulations and guidelines, and was granted by UK Health Research Authority Newcastle and North Tyneside-1 National Health Service (NHS) Research Ethics Committee (ref: 09/H0908/42). Donors had received clinical assessments during life and consented to the use of brain tissue for research purposes. At the time of death next of kin assented to tissue donation.

### Clinical information

Post-mortem tissue was obtained from the Newcastle Brain Tissue Resource (NBTR). Seventeen controls, 15 DLB cases without depression and 13 DLB cases with depression were included for immunohistochemical analysis (see Supplementary Table 1), with 12 from each group used for biochemical analysis (see Supplementary Table 2), with 1 control, 6 DLB cases without and 7 DLB cases with depression overlapping between cohorts. Inclusion criteria for depression diagnosis used the Cornell Scale for Depression in Dementia (CSDD) (score ≥ 8) as a validated rating scale. Alternatively, the Geriatric Depression Scale (GDS) (score ≥ 10) was used if CSDD scores were not available (see Supplementary Methods).

### Immunohistochemistry

Formalin fixed paraffin embedded tissue blocks from the right hemisphere containing the sgACC (Brodmann area: BA25) sampled at the rostrum of the corpus callosum were cut at 10 μm using a rotary microtome. Immunohistochemical staining used established protocols (see Supplementary Methods).

For STED microscopy, sections were blocked using 10% normal goat serum (NGS: Sigma G9023) for one hour at room temperature, followed by incubation with primary antibodies overnight at 4 °C (see Supplementary Table 3). After washes in TBS, sections were incubated with secondary fluorescent antibodies in TBS and 10% NGS for 1 h at room temperature. Nuclei were stained with TO-PRO™-3 Iodide (Invitrogen, UK) and mounted using ProLong Glass Antifade Mountant (Thermo Fisher, UK) using high precision coverslips (0.170 ± 0.01 mm thick; Roth, Germany, LH25.1).

Densitometric analysis was used to assess the percentage area stained of immunoreactivity within the region of interest (ROI) (see Supplementary Methods). An adapted stereological method was used to estimate the number of sgACC neurones (see Supplementary Methods). Midbrain sections containing the VTA and SN at the insertion of the oculomotor nerve were used to estimate pigmented neurone numbers [26].

### Estimation of dopaminergic and serotonergic fibre density

For dopaminergic and serotonergic fibre analysis in sgACC, images were captured using a microscope with a motorised stage (Zeiss, Germany) coupled to a PC. Stereologer software (Stereologer, Bethesda, MD, USA) was used to ensure adequate and unbiased sampling. The ROI was drawn at 1.25X magnification and a randomly-oriented point grid superimposed over the image. Within the ROI, 10–15 frames were captured at 10X magnification. DAT and serotonin transporter (5HTT) positive fibres within sgACC were assessed by counting the number of intersections between the linear probe and lines representing the surface feature within a dissector frame of known dimensions. The isotropic interaction between the linear probes and the surface feature was achieved by using VUR (vertical uniform random) sections in combination with cycloid sine-weighted line probes [29].

### STED analysis

Triple-colour STED microscopy was applied to assess DAT or 5HTT co-localisation with a presynaptic terminal marker (synaptosomal associated protein 25 kDa [SNAP25]) and phosphorylated α-synuclein (s129), which provides an indication of both physiological and pathological α-synuclein. STED 3D images were acquired using a Leica SP8 STED microscope and Application Suite X software (LAS X; Leica Microsystems) with 100x/1.4NA STED white oil immersion objective. Images of 256 × 256 pixels were obtained using 35x optical zoom, resulting in a pixel size of 13 × 13 nm. Images were deconvolved using Huygens Essential Software (Scientific Volume Imaging, Netherlands). The Object Analyser Advanced tool in Huygens was used to create 3D surfaces for each channel and obtain the quantitative measures of individual particles. Co-localisation measurements were used to assess spatial overlap between structures in different data channels. Synapses were defined by an overlap of greater than 80% of SNAP25 staining and DAT or 5HTT staining. Alpha-synuclein positive synapses were defined where s129 staining overlap with the synapse (SNAP25 and DAT/5HTT) exceeded over 50% of stained volume.

### Western blot and dot blot analysis

Western and dot blot analysis used established methods (see Supplementary Methods, Supplementary Table 3 and Supplementary Figs 1 and 2).

### Statistical analysis

Statistical analyses were performed using SPSS Statistics version 22.0 (see Supplementary Methods). We used a group analysis to determine the overall effects of disease and symptom, with specific within disease analysis to determine differences between depressed and non-depressed DLB donors.

## Results

### Pathology in subgenual cingulate

**Fig. 1:**
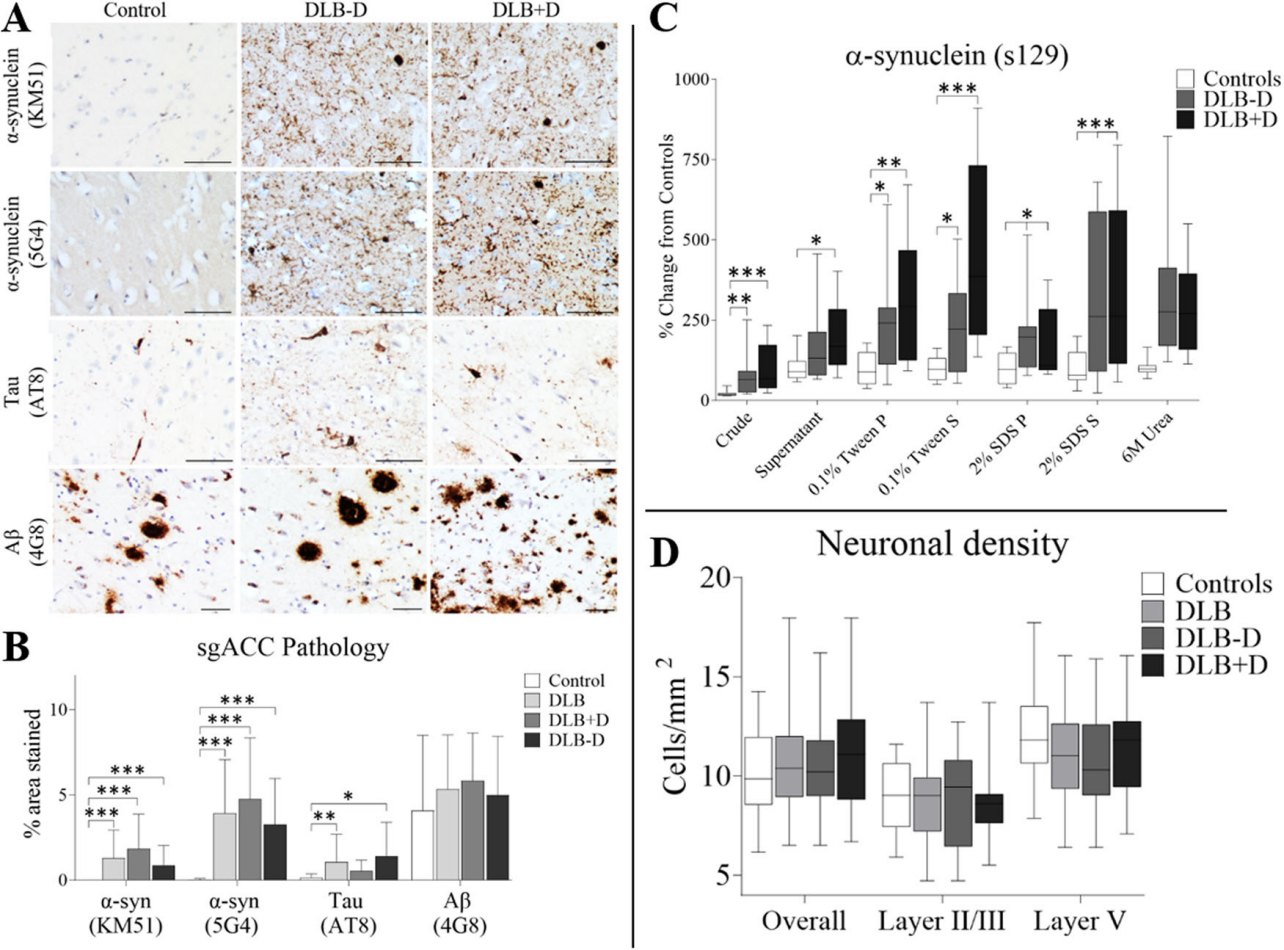
Effect of pathology on the sgACC. **A** Photomicrographs of α-synuclein (KM51), α-synuclein (5G4), Tau (AT8) and Aβ (4G8) pathology in sgACC (BA25) in controls, DLB cases with and without depression. Magnification x10; scale bars represent 100 μm. **B** α-synuclein (KM51), (5G4), Tau (AT8) and Aβ (4G8) pathology (% area stained) in sgACC in Controls (*n* = 17), DLB cases overall (*n* = 28), with (*n* = 13) and without depression (*n* = 15); (*p < 0.05, **p < 0.01 and *** *p* < 0.001, compared to control group). **C** Dot blotting of α-synuclein phosphorylated at position serine 129 in sgACC within different tissue fractions: crude, supernatant (cytoplasmic soluble proteins), 0.1% Tween pellet (soluble membrane bound proteins), 0.1% Tween supernatant (Tween soluble membrane proteins), 2% SDS pellet (insoluble membrane bound proteins), 2% SDS supernatant (SDS soluble membrane proteins) and 6 M Urea supernatant (highly insoluble proteins) Controls (*n* = 12), DLB-D (*n* = 12) and DLB + D (*n* = 12). **D** Neurones immunopositive for the neuronal marker HuD were determined in layers II/III and layer V and overall (cells per/mm^2^) within sgACC in Controls (*n* = 17), DLB cases overall (*n* = 28), with (*n* = 13) and without depression (*n* = 15); Data presented as mean and SD.

A significant main effect of diagnosis on α-synuclein pathological burden was observed in sgACC *H*(3) = 31.370, *p* < 0.001, with significant increase identified in DLB cases overall, with or without depression, compared to controls (*p* < 0.001; Fig. 1A). Alpha-synuclein (5G4) pathological burden was significantly different in sgACC between groups *H*(3) = 40.174, *p* < 0.001, with significant increases in DLB cases overall, with or without depression compared to controls (*p* < 0.001; Fig. 1B). In a paired comparison, α-synuclein pathology was not different between depressed and non-depressed DLB donors (KM51 *p* = 0.338, 5G4 *p* = 0.251, Mann Whitney U). Tau pathological burden was also significantly different between groups *H*(3) = 12.140, *p* = 0.007. DLB cases with depression showed similar p-Tau burden compared to controls, however DLB cases overall (*p* = 0.010) and DLB without depression (*p* = 0.015) showed an elevated tau burden in sgACC compared to controls. Comparing DLB cases with and without depression showed unaltered tau burden between depressed and non-depressed DLB donors (*p* = 0.459, Mann-Whitney U). Aβ burden was not significantly different between groups *H*(3) = 3.266, *p* = 0.352 (Fig. 1B) or when comparing DLB subgroups directly (*p* = 0.377, Mann-Whitney U).

### Biochemical analysis of α-synuclein

Using s129 antibody to assess biochemical changes (dot blot) using sgACC fractionated tissue [30] generally supported immunohistochemical data and showed no significant difference in α-synuclein levels between DLB cases with and without depression with the exception of the 2% SDS pellet (*p* = 0.001, Mann-Whitney U). Increased s129 was found using dot blotting compared to control in the crude sample *(H*(2) = 16.826, *p* < 0.001), supernatant (*H*(2) = 7.115, *p* = 0.029), 0.1% Tween pellet (*H*(2) = 11.988, *p* = 0.002), 0.1% Tween supernatant (*H*(2) = 16.122, *p* < 0.001), 2% SDS pellet (*H*(2) = 6.504, *p* = 0.039), 2% SDS supernatant (*H*(2) = 9.159, *p* = 0.028) and urea fraction of tissue homogenates (*H*(2) = 20.523, *p* < 0.001). DLB cases with depression showed higher s129 burden compared to controls in crude (*p* = 0.001), supernatant (*p* = 0.029), 0.1% Tween pellet (*p* = 0.003), 0.1% Tween supernatant (*p* < 0.001), 2% SDS supernatant (*p* = 0.020) and urea (*p* = 0.001). DLB cases without depression showed higher s129 levels compared to controls in the crude (*p* = 0.002), 0.1% Tween pellet (*p* = 0.045), 2% SDS pellet (*p* = 0.010) and 2% SDS supernatant (*p* = 0.035) and urea fraction (*p* < 0.001) (Fig. 1C).

The neuronal specific marker (HuD) [31] showed no significant difference in neuronal density overall *F*(2, 48) = 0.393, *p* = 0.677, or within layers II/III *F*(2, 48) = 0.018, *p* = 0.982, or layer V *F*(2, 48) = 1.183, *p* = 0.315 of the sgACC between groups (Fig. 1D).

### Dopaminergic and serotonergic fibres in sgACC

**Fig. 2:**
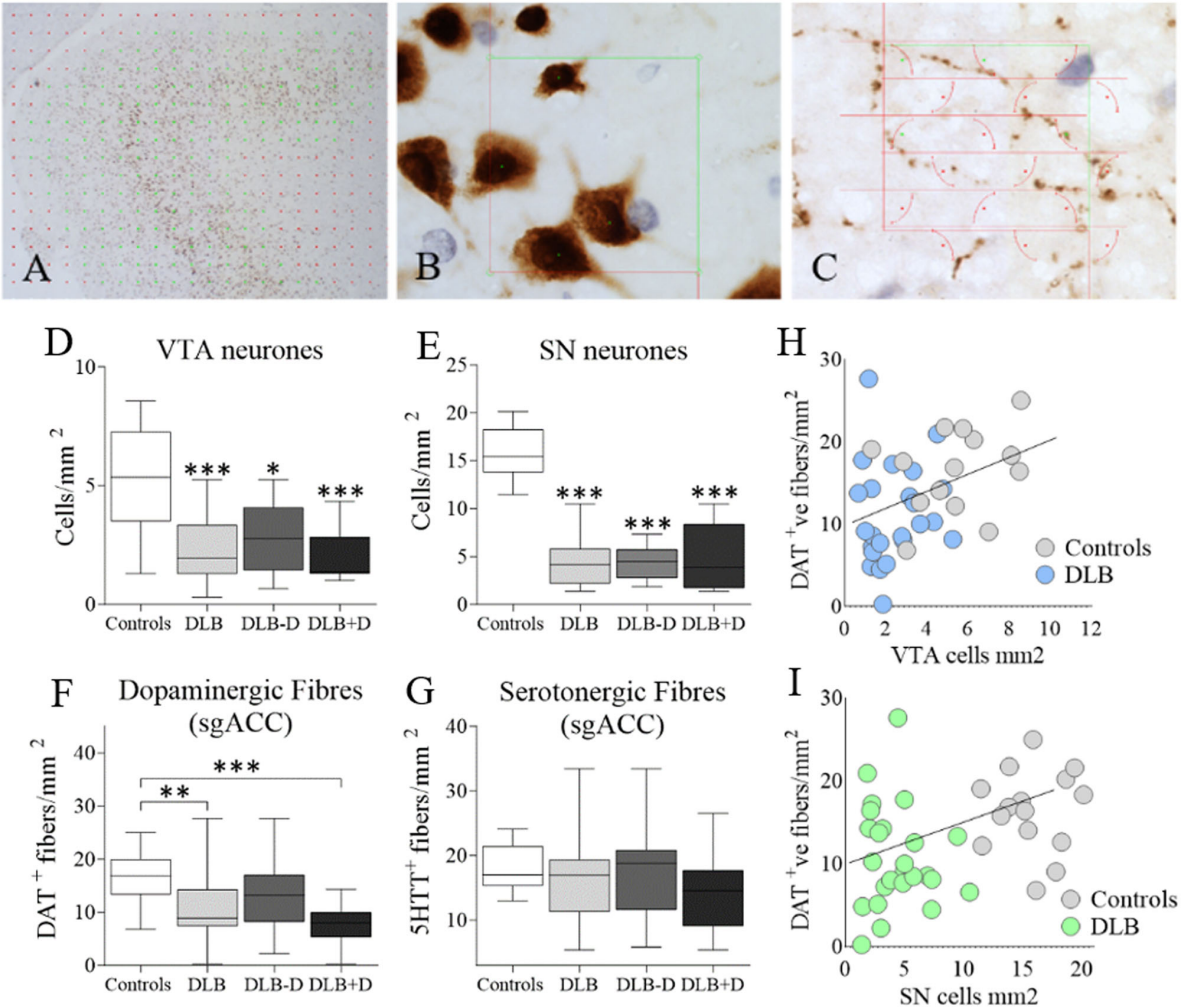
Analysis of dopaminergic cells in ventral tegmental area and substantia nigra, and dopaminergic and serotonergic fibres in sgACC. **A** For stereological imaging, a region of interest was drawn at 1.25X and a point grid was superimposed over the area, with green points representing the coordinates of sampling; **B** Randomly sampled frames in the x-y axis were taken at 63X, with neurones counted within a dissector frame of known dimensions to allow for an estimation of neurone numbers; **C** For the estimation of monoaminergic fibres within the outlined reference space, the fibres intersecting (marked green) a grid of randomly orientated cycloids were counted; **D** pigmented dopaminergic neurones in the VTA; **E** pigmented dopaminergic neurones in the SN; **F** Dopaminergic fibres (DAT positive) in sgACC; **G** Serotonergic fibres (5HTT positive) in sgACC were assessed in controls (*n* = 17), DLB cases overall (*n* = 28), DLB cases without (DLB-D) (*n* = 15) and DLB cases with depression (DLB + D) (*n* = 13) (Significant at *p < 0.05, **p < 0.01 and ***p < 0.001); Data presented as mean and SD. **H** Correlation analysis between dopaminergic neurones in ventral tegmental area (VTA) (*r*_*s*_ = 0.337, *p* = 0.007), and **I** substantia nigra (SN) (*r*_*s*_ = 0.313, *p* = 0.011) with DAT positive fibres in sgACC.

The sgACC receives dopaminergic projections from the VTA but also the SN [32–34], therefore we assessed pigmented dopaminergic neurones in the SN and VTA, along with DAT and 5HTT positive fibres in the sgACC (Fig. 2A–C). VTA neurone number was significantly different between groups *H*(3) = 19.056, *p* < 0.001, with significantly lower neuronal count in DLB overall (*p* = 0.001), DLB cases with depression (*p* = 0.001) and DLB cases without depression compared to controls (*p* = 0.035; Fig. 2D). Direct comparison of VTA neurones between depressed and non-depressed DLB donors showed a trend towards higher VTA neurone counts in non-depressed DLB donors (*p* = 0.061, Mann-Whitney U). The number of neurones in the SN was also significantly different between the groups *H*(3) = 34.179, *p* < 0.001, with significantly fewer neurones observed in all groups compared to controls (*p* < 0.001; Fig. 2E). There were no significant difference in neurone counts between depressed and non-depressed DLB donors in the SN (*p* = 0.809, Mann-Whitney U).

DAT positive fibres in the sgACC differed significantly between the groups *F*(3, 69) = 7.029, *p* < 0.001, with lower fibre density in DLB cases overall (*p* = 0.004) and in DLB cases with depression compared to controls (*p* < 0.001) but not in non-depressed DLB donors (Fig. 2F). When comparing within DLB, depressed DLB donors showed significantly lower DAT fibres compared to non-depressed donors (*p* = 0.025, Mann-Whitney U). Since abnormal serotonergic neurotransmission is implicated in depression, serotonergic innervation of sgACC was assessed using an identical approach. Serotonergic fibres were more abundant in the sgACC compared to dopaminergic fibres *χ2*(1) = 8.138, *p* = 0.004. No significant difference in the number of serotonergic fibres in the sgACC was observed between groups *F*(2, 39) = 1.694, *p* = 0.197 (Fig. 2G), or when directly comparing depressed to non-depressed DLB donors (*p* = 0.168, Mann-Whitney U). No significant correlations occurred between dopaminergic neurones in VTA or SN, and DAT positive fibre density in sgACC within the groups (Supplementary Fig. 3). In the combined control and DLB groups however, a significant correlation was observed between DAT fibre density and VTA neurones *r*_*s*_ = 0.337, *p* = 0.007, as well as SN neurones *r*_*s*_ = 0.313, *p* = 0.011 (Fig. 2H, I).

### Dopaminergic and serotonergic synapses in sgACC

**Fig. 3:**
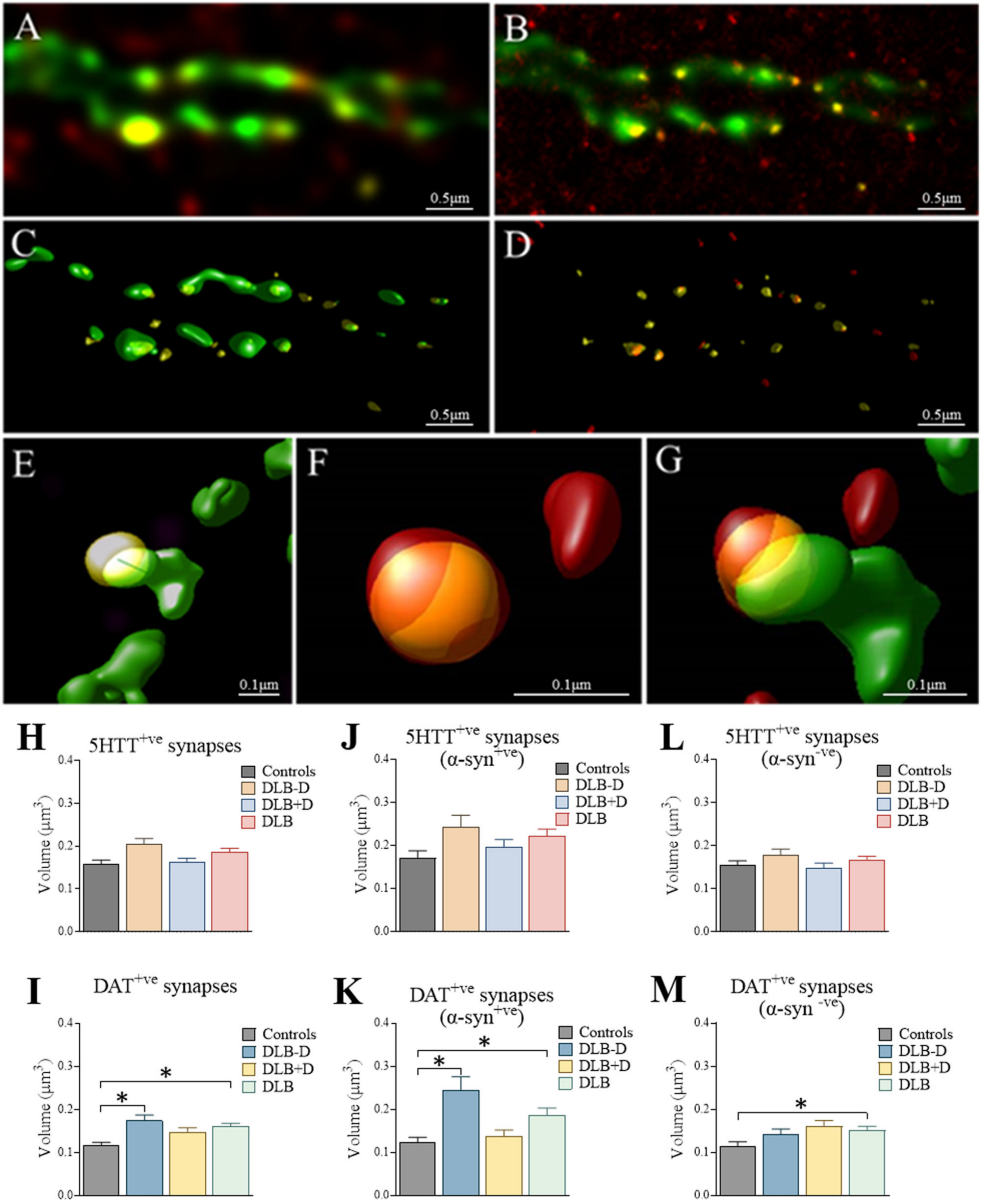
Stimulated emission depletion (STED) imaging of dopaminergic and serotonergic synapses in subgenual anterior cingulate. **A** - STED 3D image of dopaminergic fibres and terminals (DAT; green), presynaptic terminals (SNAP25; yellow) and phosphorylated α-synuclein (s129; red); **B -** Deconvolved STED image; **C**, **E -** 3D surfaces of dopaminergic fibres and presynaptic terminals; **D**, **F -** 3D surfaces of presynaptic terminals and α-synuclein; **G** - 3D surfaces of co-localisation of all 3 channels; **H** Co-localisation of serotonergic (5HTT + ve) and **I** dopaminergic (DAT + ve) synapses with pre-synaptic terminal marker (SNAP25) (>80% co-localisation); **J**, **K** Serotonergic and dopaminergic synapses α-synuclein (s129) + ve, and **L**, **M** α-synuclein (s129)–ve synapses in sgACC were assessed in controls (*n* = 17), DLB cases overall (*n* = 28), DLB cases without (DLB-D) (*n* = 15) and DLB cases with depression (DLB + D) (*n* = 13) (Significant at *p < 0.05); Data presented as mean and SEM.

Morphometric findings were extended by assessment of serotonergic (5HTT and SNAP25 positive) and dopaminergic synapses (DAT and SNAP25 positive) in relation to phosphorylated α-synuclein (s129) in the sgACC using STED (Fig. 3). Due to the high resolution analysis provided by STED microscopy, an unbiased stereological analysis was not possible.

5HTT containing synapses were not different in size between groups *H*(3) = 0.833, *p* = 0.842 or when comparing within the DLB groups (*p* = 0.386, Mann-Whitney U). No significant difference was observed in the volume of α-synuclein positive *H*(3) = 2.089, *p* = 0.554, or α-synuclein negative *H*(3) = 0.803, *p* = 0.749 serotonergic synapses between groups (Fig. 3H, J, L), or when comparing directly between DLB groups (α-synuclein positive: *p* = 0.583; α-synuclein negative: *p* = 0.667, Mann-Whitney U).

DAT synapse volume was significantly different between groups *H*(3) = 12.007, *p* = 0.007, with larger DAT synapses observed in DLB overall (*p* = 0.011), as well as non-depressed DLB cases (*p* = 0.017) compared to control (Fig. 3I). DAT positive synapse volume was not significantly different when comparing within DLB groups directly (*p* = 0.531, Mann-Whitney U). Specifically, DAT synapses containing s129 α-synuclein were larger compared to controls in the DLB group overall (*p* = 0.043), and in the non-depressed DLB group (*p* = 0.016), but not in the depressed DLB group compared to control (Fig. 3K). Direct comparison of α-synuclein positive synapses between DLB subgroups showed synapses to be smaller in depressed DLB donors compared to non-depressed donors (*p* = 0.012, Mann-Whitney U). Alpha-synuclein negative dopaminergic synapses were significantly larger in DLB cases (*p* = 0.028) compared to control α-synuclein negative synapses, but unchanged between DLB subgroups (*p* = 0.286, Mann-Whitney U; Fig. 3M).

**Fig. 4:**
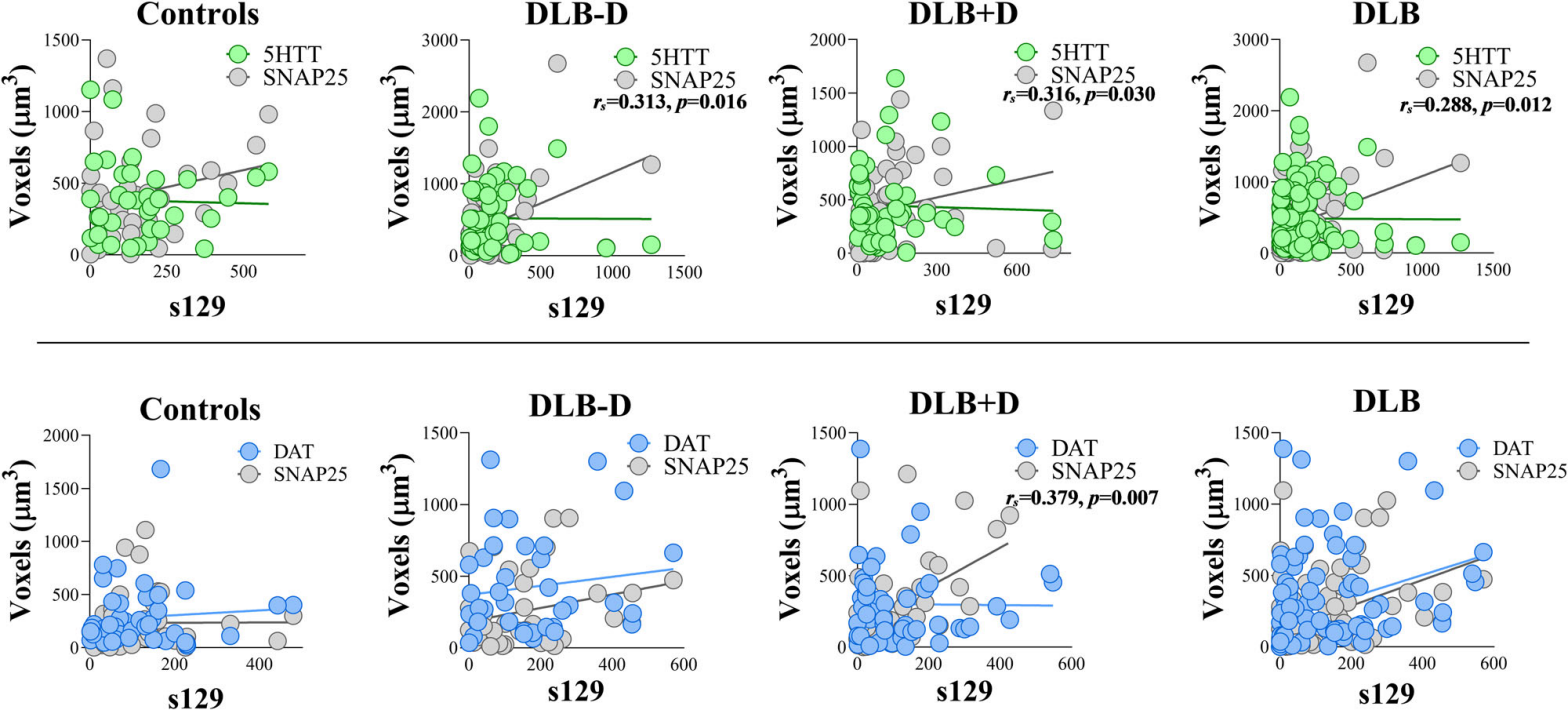
Effect of phosphorylated α-synuclein (s129) on serotonergic and dopaminergic synapses. **Top)** The relationship between 5HTT and SNAP25, and **Bottom)** DAT and SNAP25 with s129 within synapses in the sgACC was assessed using STED microscopy and Spearman’s correlation analysis in controls, DLB cases overall, DLB cases with (DLB + D) and without (DLB-D) depression.

Alpha-synuclein can interact with synaptic vesicle proteins [35], therefore we determined if α-synuclein within dopaminergic or serotonergic synapses correlated with synaptic activity assessed using SNAP25. A significant positive correlation between the volume of s129 and SNAP25 within presynaptic 5HTT terminals was observed in DLB overall (*r*_*s*_ = 0.288, *p* = 0.012), non-depressed DLB (*r*_*s*_ = 0.313, *p* = 0.016) and DLB with depression (*r*_*s*_ = 0.316, *p* = 0.030; Fig. 4). A significant positive correlation was observed between s129 volume and SNAP25 within DAT terminals in DLB cases with depression (*r*_*s*_ = 0.379, *p* = 0.007; Fig. 4), but not in DLB cases without depression or controls.

A significant correlation was observed between VTA neurones and the volume of DAT synapses in sgACC in DLB cases with depression (*r*_*s*_ = 0.710, *p* = 0.049; Supplementary Fig. 4). Furthermore, a significant correlation in DLB cases with depression was observed between VTA neurone number and volume of α-synuclein positive DAT synapses (*r*_*s*_ = 0.732, *p* = 0.016; Supplementary Fig. 5). No significant correlations were observed between SN neurones and DAT positive synapse volume in sgACC within groups (Supplementary Fig. 4).

### Monoaminergic protein analysis

**Fig. 5:**
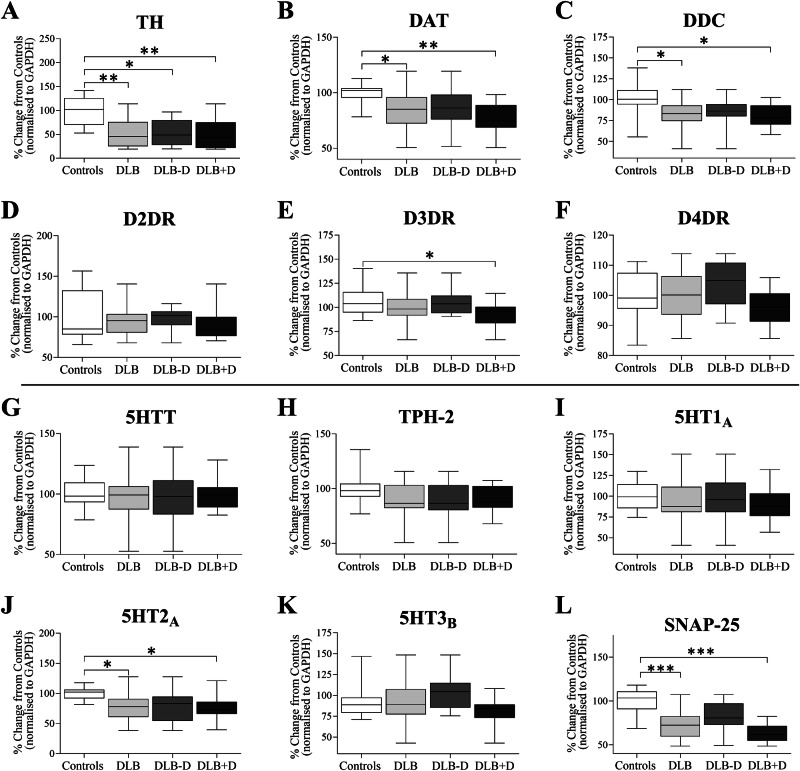
Dot blot analysis of dopaminergic and serotonergic markers and receptors in sgACC. Dopaminergic markers, including **A** tyrosine hydroxylase (TH), **B** dopamine transporter (DAT), **C** dopamine decarboxylase (DDC), and **D**–**F** dopamine receptors D2, D3 and D4, as well as serotonergic markers, including **G** serotonin transporter (5HTT), **H** tryptophan hydroxylase-2 (TPH-2), and **I**–**K** serotonin receptors 5HT1_A_, 5HT2_A_ and 5HT3_B_, and **L** SNAP-25 were assessed in sgACC in age-matched controls (*n* = 12), DLB cases overall (*n* = 24), DLB cases with (DLB + D) (*n* = 12) and without (DLB-D) depression (*n* = 12); (*p < 0.05, **p < 0.05 and ***p < 0.001, compared to appropriate group); data are presented as mean and SD.

To determine the impact of DLB on dopaminergic and serotonergic markers we used western blotting. DAT *F*(3, 56) = 4.346, *p* = 0.008, tyrosine hydroxylase (TH) *F*(3, 56) = 8.762, *p* < 0.001, dopamine decarboxylase (DDC) *F*(3, 56) = 3.660, *p* = 0.018 and dopamine D3 receptor (D3DR) levels *F*(3, 56) = 2.913, *p* = 0.042 were significantly different between the groups in the sgACC (Fig. 5A–F). DLB cases overall showed significantly lower DAT (*p* = 0.022), and in depressed DLB donors compared to control (*p* = 0.009), but not comparing depressed against non-depressed DLB donors using direct comparison (*p* = 0.219, Mann-Whitney U). TH protein was reduced compared to control in DLB overall (*p* = 0.002) and in depressed (*p* = 0.003) or non-depressed donors (*p* = 0.012) compared to controls, but not between depressed and non-depressed donors when compared directly (*p* = 0.756, Mann-Whitney U). Lower DDC levels were seen in DLB overall compared to controls (*p* = 0.024), and in DLB cases with depression compared to controls (*p* = 0.041), but there was no change in DDC seen between depressed and non-depressed DLB (*p* = 0.410, Mann-Whitney U). No significant difference was observed in dopamine D2 receptors (D2DR) *F*(3, 56) = 0.620, *p* = 0.605 between groups. Significantly lower D3DR levels were observed in the DLB with depression when compared with control (*p* = 0.049). When directly comparing DRD3 protein between depressed and non-depressed DLB donors, DRD3 protein was significantly reduced in depressed donors (*p* = 0.045, Mann-Whitney U). Although no significant change was seen in D4 receptors (D4DR) *F*(3, 56) = 2.115, *p* = 0.109 between groups, a reduction was seen in D4DR in DLB donors with depression when directly compared to non-depressed DLB donors (*p* = 0.020, Mann-Whitney U).

Assessing the serotonin system in DLB, no significant changes in 5HTT *F*(3, 56) = 0.064, *p* = 0.978 were seen between groups, or in a direct comparison of depressed and non-depressed DLB donors (*p* = 0.799, Mann-Whitney U). Tryptophan hydroxylase 2 (TPH-2) *F*(3, 56) = 1.719, *p* = 0.173 was also unaltered between groups and when comparing directly within DLB groups (*p* = 0.977, Mann-Whitney U). Serotonin 1 _A_ (5HT1_A_) receptor *F*(3, 56) = 0.258, *p* = 0.855 expression was also unaltered when comparing groups overall or within DLB groups directly (*p* = 0.773, Mann-Whitney U). Serotonin 3_B_ (5HT3_B_) receptor showed a trend towards alterations between groups *F*(3, 56) = 2.156, *p* = 0.062, with a significant reduction in 5HT3_B_ protein in DLB donors with depression when compared directly with non-depressed DLB donors (*p* = 0.017, Mann Whitney U). A significant difference in 5HT2_A_ receptor protein levels was observed in the sgACC between groups *F*(2, 34) = 3.858, *p* = 0.019, with significantly lower 5HT2_A_ observed in DLB cases overall (*p* = 0.026) and in DLB cases with depression compared to controls (*p* = 0.044), but not when comparing DLB donors with or without depression directly (*p* = 0.590, Mann-Whitney U; Fig. 5G–L).

**Fig. 6:**
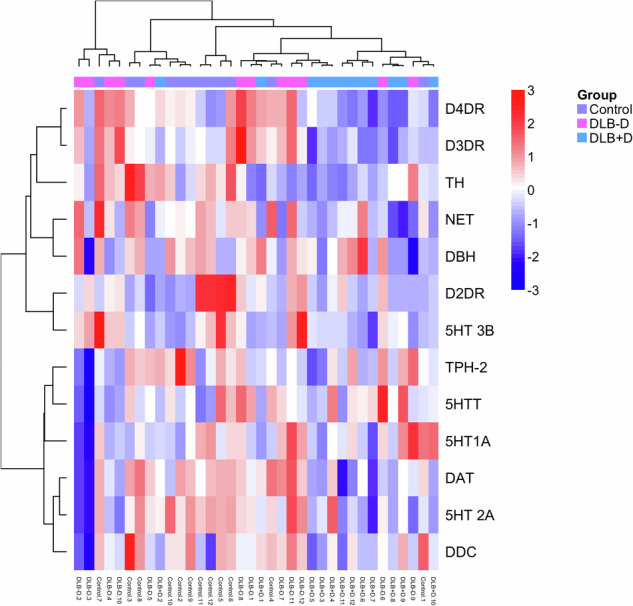
Heat map analysis of monoaminergic markers and receptors in the sgACC. Hierarchical clustering analysis was performed based on monoaminergic protein relative expression in sgACC in controls (*n* = 12), DLB cases with (DLB + D) (*n* = 12) and without (DLB-D) depression (*n* = 12).

Heat map analysis showed no clear separation of disease groups based on monoaminergic proteins (Fig. 6). Using linear discriminant analysis to separate cases based on monoaminergic markers to determine the greatest influence on depression showed DAT (Wilks’ Lambda, 0.535, *p* = 0.0004) and D4DR (Wilks’ Lambda 0.442, *p* = 0.0002) in combination had greatest predictive values, showing 78% accuracy in re-classifying the original data.

## Discussion

DLB patients are at high risk of developing major depression, with depression having a significant impact on quality of life [3]. The sgACC in depression in DLB correlates with α-synuclein pathology in late onset MDD, indicating that α-synuclein may be a factor in MDD development in older individuals [36], intimately involved in the aetiology of depression [10, 27] and a high α-synuclein pathological burden in DLB [28]. We found no significant difference between depressed and non-depressed DLB donors for α-synuclein pathology suggesting gross pathology within the sgACC is not a significant driver of depressive symptoms. Significant neurone loss within the sgACC in DLB is not apparent, and there is no association with depression. Although the basis for depression in DLB is complex, our results indicate reduced dopaminergic innervation in sgACC contributes to depression. This may be specific to the cingulate cortex since assessment of dopaminergic proteins in the fusiform gyrus which is involved in visual imagery does not show major change in DLB (Gliaudelytė, unpublished). This may warrant clinical trials of D2 dopamine receptor agonists since pramipexole and pergolide show efficacy in reducing depression in patients with PD [37, 38], as well as symptoms of major depression in patients without PD [39, 40].

The mesolimbic dopamine pathway from the VTA to cortical and subcortical regions includes the cingulate with additional projections from the dorsal tier of the SN [34]. VTA dopaminergic neurones play a role in reward and stress [20] with dopaminergic dysfunction contributing to anhedonia in MDD [21]. We identified a decrease in VTA cell number in DLB cases overall, but no significant loss in relation to depression in DLB. Compared to the loss of neurones in the SN [26], the VTA shows relative preservation in PD [26, 41]. Similar to our findings, VTA cell loss and gliosis is associated with the presence of depression in PD [42, 43]. In PD, PDD, and DLB, higher midbrain α-synuclein burden associates with depressive symptoms [26] suggesting that mesolimbic and mesocortical projections contribute to depression in DLB, similar to PD [44].

We observed reduced sgACC dopaminergic fibre density in DLB cases with depression. In PD, motor symptoms appear when around 50% of SN dopaminergic neurones are lost [45], although striatal innervation is severe with around 80% terminal loss [46, 47]. The enhanced dopaminergic fibre loss seen in this study in DLB cases (~50%) with depression in the absence of severe (~20%) VTA neuronal loss may represent a similar effect with axon and terminal loss prior to major cell death.

Our STED analysis showed phosphorylated α-synuclein within sgACC dopaminergic synapses and increased phosphorylated α-synuclein in sgACC tissue homogenates. In depressed DLB cases, elevated synaptic α-synuclein corresponded with elevated SNAP25, a finding also seen with serotonergic terminals. One possibility is that pathological α-synuclein sequesters SNAP25 within synapses leading to synaptic dysfunction. Alpha-synuclein modulates synaptic activity by assisting with SNARE complex formation [35, 48], and our finding may represent a synaptic response to maintain normal neurotransmission following reduction in cell numbers [49]. Fibrillar α-synuclein can however, promote aberrant synaptic activity [50], and by forming synaptic aggregates, fibrillar α-synuclein may cause depletion of functional SNARE proteins, reducing the effective synaptic vesicle pool [51]. This may underscore the increase in SNAP25 within α-synuclein containing dopaminergic synapses, with SNAP25 increased, but non-functional due to sequestration in α-synuclein aggregates [52]. The combined effects of dopaminergic synapse reduction due to cell loss and reduced synaptic efficiency in remaining synapses may cause an effective depletion of dopamine to the sgACC cortex and contribute to depressive symptoms.

A loss of dopaminergic proteins was seen in DLB cases with depression compared to controls, including DAT, TH and DDC. Using the linear discriminant analysis, DAT and DRD4 showed significant associations with depression in DLB. These reductions align with DAT fibre loss in depressed DLB cases and reinforce the general loss of dopaminergic innervation in DLB, but particularly in DLB experiencing depression [25, 26]. Dopaminergic deficits including reduced striatal DAT binding has been observed in MDD [22] and in PD and DLB, with DLB cases showing greater caudate DAT loss compared to PD [53], and reduced ACC DAT in DLB [54]. The use of dopamine agonists in MDD can have significant benefits and may be beneficial in DLB if suitable treatment regimens are identified [55, 56].

Our results indicate minimal changes in post-synaptic D2DR in DLB, but reduced DRD3 expression. D3DR are expressed pre- and postsynaptically and have the highest affinity for dopamine [57]. DRD3 is downregulated in MDD [58], and reduced in the ventral striatum in PD cases [59], but with either downregulation [60] or no change [61] in DLB. Our results indicate that reduced DRD3 levels may play a role in depression in DLB, and DRD3 specific dopaminergic agents may be beneficial. Pramipexole and ropinirole improve depressive symptoms in PD and may provide benefit for depression in DLB, although careful monitoring may be needed [55, 62, 63]. In PD, improved working and episodic memory occurs with dopamine agonists, suggesting that dopaminergic agonists may additionally improve cognition [64, 65].

Our findings show no significant changes in sgACC DRD4 levels in DLB despite an association based on hierarchical clustering. DRD4 shows the lowest levels of expression of dopamine receptors in the brain [66]. Similar to previous studies indicating that DRD4 does not modulate depressive-like behaviours [67], our findings suggest that DRD4 may not be a treatment target.

In contrast to dopaminergic changes, serotonergic involvement in depression in DLB was limited, comparable with similar studies [43, 68]. Whilst the dorsal raphe nucleus (DRN) shows LB pathology in DLB and PD, studies of neurone loss are variable with reduction [69] or no change in DRN neurones in PD or PDD [68, 70], with neuronal loss in the DRN in DLB [71]. Due to the tissue sampling strategy and differences in DRN projection sites [72], we did not assess DRN neurone numbers. We however found no change in sgACC serotonergic fibre density in DLB similar to findings in the amygdala and dorsal prefrontal cortex in LBD donors with and without depression [68]. We did however find α-synuclein associated with serotonergic synapses and a positive correlation between SNAP25 volume and α-synuclein volume. This may indicate 5HT terminal dysfunction but unchanged innervation in the sgACC since we saw no change in fibre density or 5HTT protein levels. Other studies have also shown no association with 5HTT levels with depression or anxiety in PD [73]. Future studies should however determine the effects of α-synuclein pathology in DLB in the brainstem and midbrain serotonergic system using appropriate stereological approaches.

There has been considerable in vivo use of 5HTT ligands in PD and to a lesser extent DLB. In drug naïve PD, reductions in [(11)C-N,Ndimethyl-2-(-2-amino-4-cyanophenylthio)-benzylamine (DASB)] retention in apathetic patients are seen in the sgACC suggesting reduced serotonergic innervation [74]. Similarly, reduced DASB retention is seen in caudate and ACC in early-stage PD and throughout the disease course [75–78]. In depressed PD patients however, increased DASB retention was observed in several cortical areas although no change in DASB retention is seen in ACC in MDD [79, 80]. The combined DAT and 5HTT ligand ^123^I-N-ω-fluoropropyl-2βcarbomethoxy-3β-(4-iodophenyl) nortropane (^123^I-FP-CIT) has been used in DLB where the assumption is that cortical retention only represents 5HT innervation despite DA terminals being present. Studies with ^123^I-FP-CIT in DLB have shown either reduced cortical retention [81–83], or no cortical changes [84]. No reduction in 5HTT was seen in the ACC at post mortem in PDD, although regions such as caudate showed reductions [85]. Similarly, in DLB 5HTT assessed with cyanoimipramine showed preserved cortical binding in depressed DLB donors [86, 87]. Whilst these prior studies have shown variable outcomes, our findings indicate 5HTT reductions in DLB are mild and contributions to development of depression are limited.

Decreased serotonin 5HT1_A_ binding is indicated in the ACC in MDD [14] however, we observed no alteration of 5HT1_A_ protein levels in DLB. This contrasts with findings using the 5HT1_A_ ligand 8-Hydroxy-2-Dipropylaminotetralin showing increased binding but altered K_D_ in DLB and PD [88]. This may suggest a change in receptor occupancy due to changes in protein conformation. However, our finding of decreased 5HT2_A_ protein in sgACC in DLB with depression corresponds with reduced 5HT2_A_ binding in ACC and DLPFC in MDD [14] and in DLB and PDD [89, 90]. These changes in 5HT2_A_ but not 5HT1_A_ suggests region and receptor specific changes that may benefit from selective targeting. Pimavanserin, a 5HT2_A_ selective inverse agonist and antagonist, has been used in psychosis treatment in PD [91, 92]. Despite mixed benefits in MDD [93, 94], use of Pimavanserin for depression in PD in an 8-week trial showed improved mood [95], although longer-term use of Pimavanserin may show reduced efficacy [96]. The possibility of using Pimavanserin in the treatment of depression may be warranted in DLB [97].

A limitation of this study is that while the effect of phosphorylated α-synuclein on monoaminergic synapses was explored, future studies should focus on how different α-synuclein species affect synaptic function in DLB using approaches such as cell culture and seeded aggregation assays. As with many post mortem studies, assessment is made at the end of the disease, and extrapolation to early disease stages is a limitation. Whilst our study used a generalised assessment of depression in donors, longitudinal assessment of depression using unified and dementia-appropriate depression scales should be used alongside in vivo imaging to determine changes in monoamine function. Subsequent analysis would provide a clear definition of the effects of neurochemistry on depression throughout the disease course. Studies with larger donor cohorts that address gender differences, variability in timing, severity, duration and treatment of depression in DLB would likely further current understanding in the pathophysiology of depression in DLB. Studies involving other brain regions and neurotransmitter systems, particularly a deeper evaluation of the serotonergic system involving counts of raphe neurones would also further current understanding of anatomical, functional, metabolic and neurochemical changes in relation to depression in DLB.

In summary, our results suggest that there is primarily reduced dopaminergic innervation in the sgACC in DLB cases with depression with relatively preserved dopaminergic innervation found in non-depressed DLB donors. Depressed DLB donors showed reduced levels of dopaminergic markers and receptors, and changes in dopaminergic synaptic function with maintenance of dopaminergic function observed in non-depressed DLB donors. Careful treatment with selective dopaminergic agonists or positive allosteric modulators may be beneficial in alleviating depressive symptoms in DLB.

## Supplementary information

Supplementary Methods

## Data Availability

All data will be made available upon reasonable request to the authors.
