# Supplementary material for: Dopaminergic changes in the subgenual cingulate cortex in dementia with lewy bodies associates with presence of depression
Source: Transl Psychiatry. 2025 Mar 20;15:89. doi: 10.1038/s41398-025-03298-3 (PMC11926374; doi:10.1038/s41398-025-03298-3)
Supplement: Supplementary file 1 — Supplementary Methods [file 41398_2025_3298_MOESM1_ESM.docx]

Dopaminergic Changes in the Subgenual Cingulate Cortex in

Dementia with Lewy Bodies Associates with Presence of Depression

Lina Gliaudelytė, PhD^1^*, Steven P Rushton, PhD^2^, Rolando Berlinguer-Palmini, PhD^3^, Alan J Thomas, PhD^1,4,5,6^, Christopher M Morris, PhD^1,6^.

^1^Alzheimer’s Society Doctoral Training Centre, Edwardson Building, Newcastle University, NE4 5PL

^2^School of Natural and Environmental Sciences, Agriculture Building, Newcastle University, NE1 7RU

^3^Bioimaging Unit, Leech Building, Newcastle University, Framlington Place, NE2 4AA

^4^Biomedical Research Building, Newcastle University, NE4 5PL

^5^Queen Elizabeth Hospital, Queen Elizabeth Avenue, Gateshead, Tyne and Wear, NE9 6SX

^6^Newcastle Brain Tissue Resource, Edwardson Building, Newcastle University, NE4 5PL, UK

**Supplementary Material**

# Supplementary Methods

1.1 Clinical Cohort

1.2 Immunohistochemistry

1.3 Estimation of Pathology Load

1.4 Analysis of Neuronal Density

1.5 Western Blot and Dot Blot analysis

1.6 Statistical analysis

# 2. Supplementary Tables

- 1. Supplementary Table 1. Demographic Information for Biochemical Cohort
  2. Supplementary Table 2. Demographic Information for Immunohistochemical Cohort
  3. Supplementary Table 3. Antibodies used in the study.

# 3. Supplementary Figures

- 1. Supplementary Figure 1. Western blots showing the specificity of the antibodies
  2. Supplementary Figure 2. s129 α-synuclein protein levels in tissue fractions of sgACC.
  3. Supplementary Figure 3. Effect of dopaminergic neurons in VTA and SN on dopaminergic fibers in sgACC.
  4. Supplementary Figure 4. Effect of dopaminergic neurons in VTA and SN on dopaminergic synapses in sgACC.
  5. Supplementary Figure 5. Effect of dopaminergic neurons in VTA and SN on α-synuclein positive dopaminergic synapses in sgACC.

1. Supplementary Methods

**1.1 Clinical Cohort**

All post-mortem human brain tissue was obtained from the Newcastle Brain Tissue Resource (NBTR). Ethical approval for the study was granted by Newcastle and North Tyneside-1 National Health Service (NHS) Research Ethics Committee (ref: 09/H0908/42). All participants had received clinical assessments during their life and had consented to the use of their brain tissue for research purposes. Neuropathological assessment was according to standardised diagnostic procedures and with clinical data was used to make a clinico-pathological diagnosis ^1-3^. A total of 17 controls, 15 DLB cases without depression and 13 DLB cases with depression were included in the pathological cohort (see Supplementary Table 1), with 12 from each group used for biochemical analysis (see Supplementary Table 2). Four DLB cases also fulfilled the neuropathological criteria for high AD neuropathological change and could therefore be classified as neuropathologically mixed DLB/AD with a Lewy body disease (LBD) clinical phenotype ^4, 5^. All cases were matched as closely as possible for age, sex and post-mortem delay (Supplementary Tables 1 and 2). For immunohistochemical analysis the cases were selected with shorter time in fixative, as prolonged time in fixative may diminish antigenicity and IHC efficacy in brain tissue. For biochemical analysis the cases were selected with higher pH (as a surrogate for agonal state), as low pH signals limitations for post mortem investigations ^6^. The inclusion criteria for depression diagnosis was made using the Cornell Scale for Depression in Dementia (CSDD) (score≥8) as a validated rating scale ^7^. Alternatively, the Geriatric Depression Scale (GDS) (score≥10) was used when the CSDD was not available. This has been shown to have acceptable qualities when applied to demented elderly patients ^8^. Ten DLB cases with depression had received treatment during life with selective serotonin reuptake inhibitors (SSRIs), and two cases with serotonin and noradrenaline reuptake inhibitors (SNRIs).

**1.2 Immunohistochemistry**

Following paraffin wax removal through xylene, sections were rehydrated in decreasing concentrations of ethanol. Citrate buffer heat-induced antigen retrieval (10min, 0.1M, pH 6.0) was used, followed by incubation with 3% hydrogen peroxide for 20 minutes to quench peroxidase activity. Tissue sections were incubated with primary antibodies at 4°C overnight (see supplementary Table 3). Following washes in 10mM tris-buffered saline containing 0.1% v/v Tween 20 (TBST) pH 7.6, sections were incubated with horseradish peroxidase (HRP) polymer conjugated Universal Probe for 30 minutes at room temperature, followed by incubation for 30 minutes with HRP reagent (MenaPath, Menarini Diagnostics UK). Visualisation was performed using diaminobenzidine substrate (Menarini X-Cell-Plus HRP Detection Kit; Menarini Diagnostics UK), for two minutes. Sections were counterstained with Mayer’s haematoxylin and mounted using DPX mounting medium (Cell Path, UK).

**1.3 Estimation of Pathology Load**

Densitometric analysis was used to assess the percentage area stained of immunoreactivity within the region of interest. The images were captured at 10X magnification and imported into Fiji Image J analysis software (Windows 64-bit: https://fiji.sc) for densitometric analysis (Figure 2 D-F). The Red-Green-Blue (RGB) thresholds were adjusted manually for each antibody to eliminate the detection of non-specific background staining. The percentage area stained for each antibody was quantified overall, as well as in cortical layers II, III and V. The mean percentage area stained per case was calculated from the mean values obtained across all images taken.

**1.4 Analysis of Neuronal Density**

An adapted stereological method was used to estimate the number of neurons within the sgACC. Within each coronal section, a region of interest was drawn at low magnification (1.25X) using the Zeiss Z1 microscope with a motorised stage. A randomly oriented point grid was superimposed over the observed image ensuring the sampling of the structure in a systematic and unbiased manner through its x-y axis. The distance between points on the grid was determined and HuD positive neurons were counted within a disector frame of known dimensions using a 63X oil immersion objective. The neuronal density within the sgACC was determined automatically by the Stereologer software (cells per µm^2^), with values converted to cells per mm^2^.

**1.5 Western Blot and Dot Blot analysis**

For monoaminergic marker analysis, approximately 50mg unfixed frozen grey matter from the left hemisphere corresponding to sgACC was homogenised using a rotor-stator homogeniser in ice cold lysis buffer consisting of 0.2M triethylammonium bicarbonate (TEAB) pH 7.2 (Sigma-Aldrich, MO, USA), and 1X EDTA free protease inhibitor cocktail (Complete, Roche, UK). Protein concentration in samples was determined using Bradford assay^9^ against standards of known protein concentration prepared using bovine serum albumin (BSA; Sigma-Aldrich, UK).

For phosphorylated α-synuclein analysis, total protein homogenate was fractionated to extract soluble and insoluble proteins ^10^. The soluble protein fraction containing supernatant was extracted following centrifugation (20,000 x g for 45min at 4°C), and the pellet re-suspended in 500μl of 0.1% Tween20 in 0.2M TEAB. The soluble membrane bound protein fraction was stored at -30^o^C. The remaining sample was centrifuged to extract Tween20 soluble membrane bound proteins. The pellet was re-suspended in 500μl of 2% sodium dodecyl sulphate (SDS), and the insoluble membrane bound protein fraction was stored at -30^o^C. The remaining sample was centrifuged further to extract SDS soluble membrane bound proteins, and the pellet re-suspended in 500μl of 6M Urea. The highly insoluble protein fraction was taken before further centrifugation to extract highly insoluble aggregated proteins. All protein fractions were stored at -30^o^C prior to use.

Protein samples for dot blot were prepared at 1 µg/µl with 4X Orange G Loading Buffer, 10X NuPAGE Sample Reducing Agent (Invitrogen) and homogenising buffer (0.2M TEAB). Protein standards ranging from 0 μg/μl to 120 μg/μl were prepared using pooled samples from all groups. The samples were denatured at 70°C for 10 minutes. A vacuum-assisted 96-well dot blot apparatus (Hoefer Scientific) was used to blot the samples onto nitrocellulose membrane (Amersham^TM^, 0.2μm NC) using 50µl of sample or standards per well in duplicate. The NC membrane was fixed in 70% methanol for 20 minutes under agitation and blocked using Odyssey blocking buffer (LI-COR), then incubated overnight with primary antibodies diluted in Odyssey blocking buffer and 0.2% Tween20 (Sigma-Aldrich) at 4°C. Following washes, the membranes were incubated with IRDye 800WC secondary antibodies for 1 hour at RT, then incubated with IRDye conjugated GAPDH 680nm (Santa Cruz Biotechnology) for 1 hour at RT. Each membrane was scanned at 700nm (GAPDH) and 800nm (protein of interest) for 2 minutes (LICOR-Fc) (see Supplementary Figure 1). The protein intensity bands were quantified using Image Studio Lite (LI-COR). The Lane Normalisation Factor (LNF) was calculated by dividing GAPDH signal for each lane by the highest GAPDH signal. The signal for the protein of interest was then divided by the LNF to normalise the protein of interest.

For SDS polyacrylamide gel electrophoresis (SDS-PAGE), denatured (70°C for 10min) protein samples (1 µg/µl) were loaded onto a NuPAGE 4-12% Bis-Tris Gel (Invitrogen) along with Chameleon 800 pre-stained protein ladder (LI-COR). The gel was electrophoresed in 1X NuPAGE MOPS SDS running buffer with antioxidant (Invitrogen) at 120V for 20 minutes, followed by 160V for one hour. The transfer of proteins to a nitrocellulose membrane was performed using an iBlot2 device (Invitrogen) at 20V for 1min, 24V for 4min and 27V for 5min. Blotted proteins were determined using specific antibodies (see Supplementary Figure 2).

**1.6 Statistical analysis**

Statistical analyses were performed using SPSS Statistics version 22.0. The normal distribution across samples was assessed using Shapiro–Wilk test, with homogeneity of variance determined using Levene’s test. If the assumptions of normality were met, analysis of variance (ANOVA) was used to analyse the data sets between the groups, followed by Bonferroni post-hoc analysis to correct for multiple comparisons. Where normal distribution criteria was not fulfilled, a non-parametric Kruskal-Wallis test was used to compare multiple groups, with adjusted *p*-values to correct for multiple comparisons, so that the overall error rate remained at 5%. Friedman’s ANOVA was used for pairwise comparisons within groups. Correlation analyses were carried out using Spearman’s correlation coefficient ρ (rho) (see Supplementary Figures 3, 4, and 5). Chi-square statistic was used to compare monoaminergic fibre abundancy in the sgACC. Linear discriminant analysis was used to investigate whether separation of groups could be defined on the basis of any of the monoaminergic protein markers. Based on sample size calculations using Cohen’s recommendations, a change of 30% could be detected with power of 0.96 at alpha=0.05 in a given sample with equal group sizes of 12 per group.

1. Supplementary Tables
   1. **Supplementary Table 1.** Demographic Information for Immunohistochemical Cohort


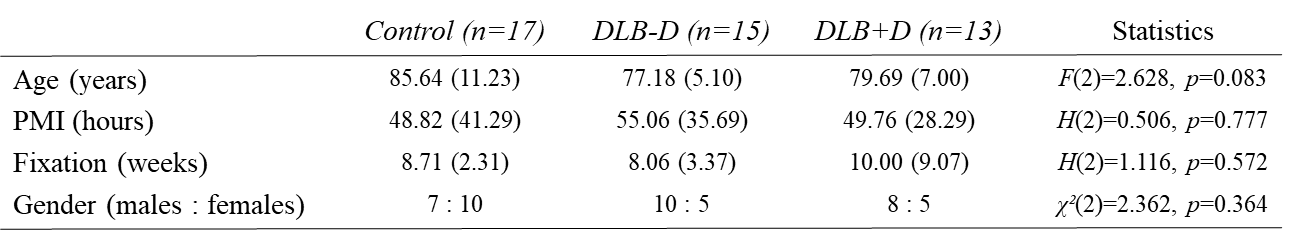
DLB without depression (DLB-D), DLB with depression (DLB+D); Age analysis (ANOVA), PMI and Fixation (Kruskal-Wallis analysis), Gender (Chi-squared test).

- 1. **Supplementary Table 2.** Demographic Information for Biochemical Cohort


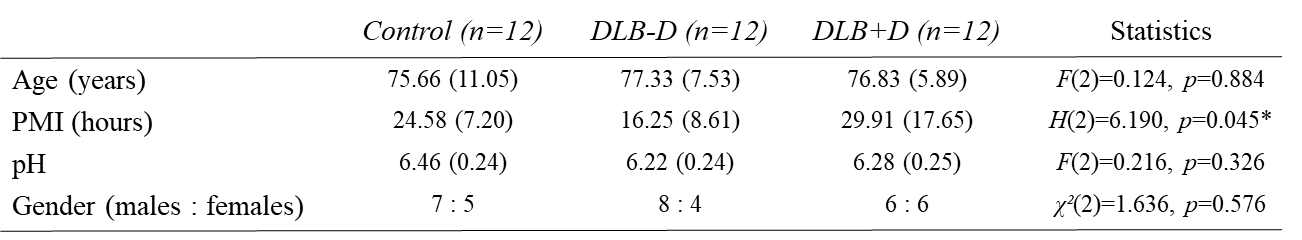
DLB without depression (DLB-D), DLB with depression (DLB+D); Age and pH analysis (ANOVA), Post-mortem Interval (PMI, Kruskal-Wallis analysis), Gender (Chi-squared test). * PMI not significant between groups after post-hoc analysis.

- 1. **Supplementary Table 3**. Antibodies used in the study.


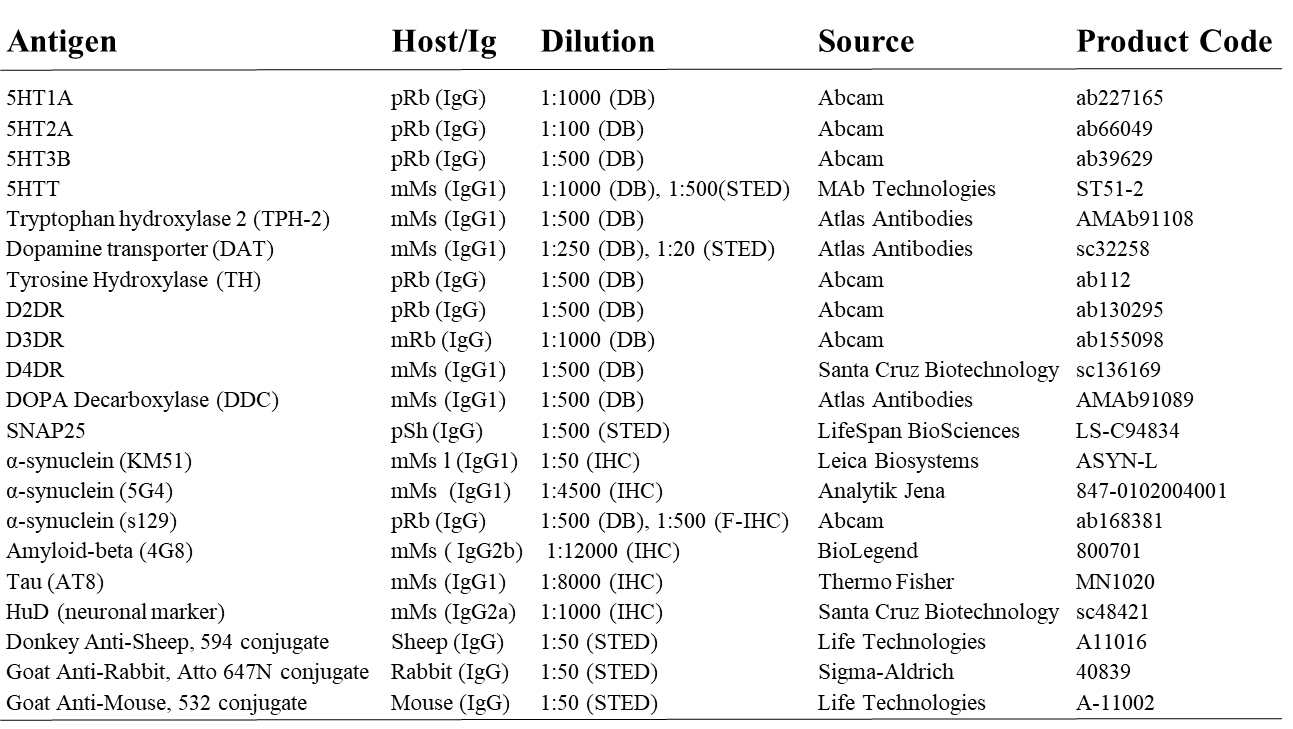
DB – dot blot; IHC – immunohistochemistry; m – monoclonal; p – polyclonal; Ms – mouse; Rb – rabbit, Gt – goat, Dk – donkey.

1. Supplementary Figures


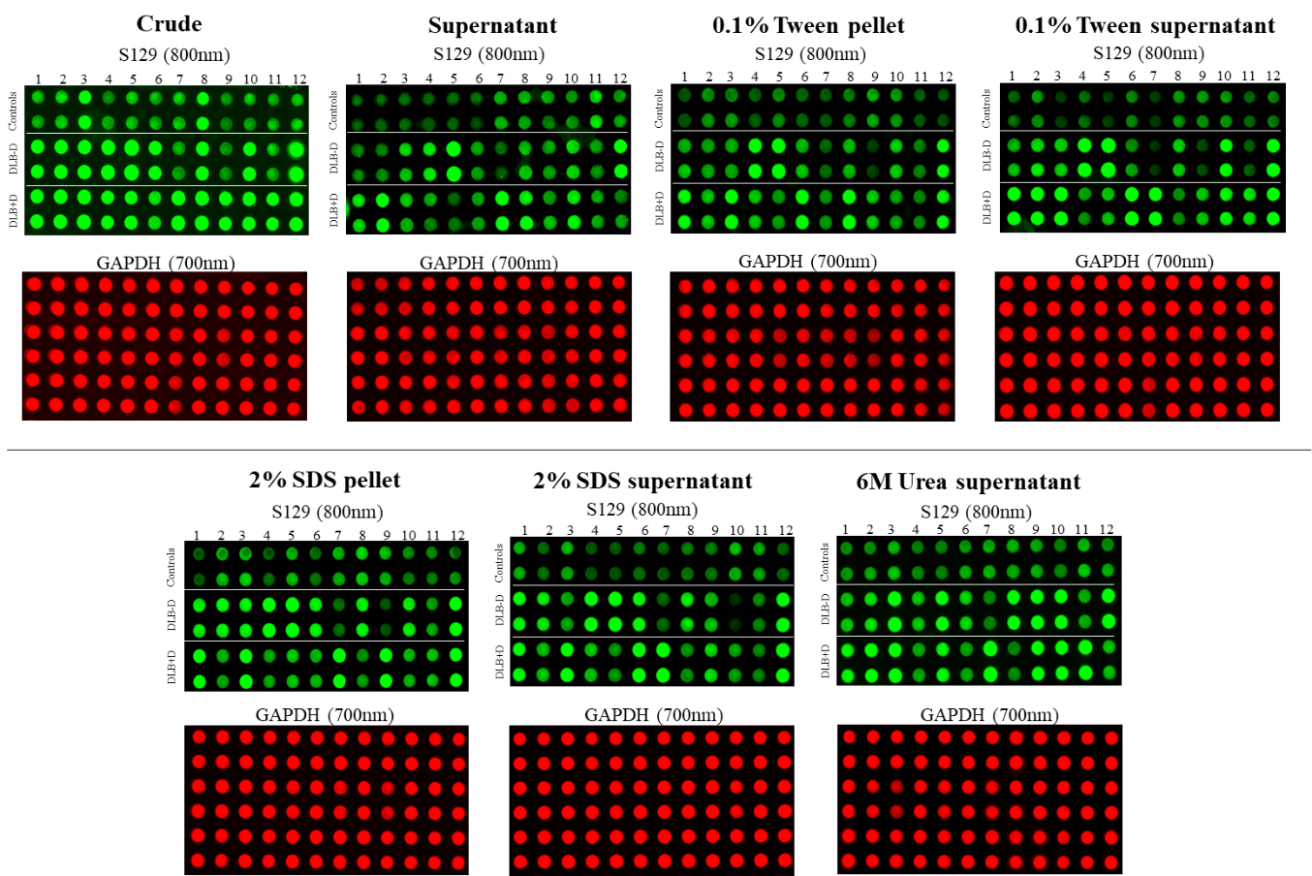


- 1. **Supplementary Figure 1.** s129 α-synuclein protein levels in tissue fractions of sgACC.

s129 in sgACC assessed within different tissue fractions of Controls, DLB cases without (DLB-D) and with depression (DLB+D): crude, supernatant (cytoplasmic soluble proteins), 0.1% Tween pellet (soluble membrane bound proteins), 0.1% Tween supernatant (Tween soluble membrane proteins), 2% SDS pellet (insoluble membrane bound proteins), 2% SDS supernatant (SDS soluble membrane proteins) and 6M Urea supernatant (highly insoluble proteins). The membranes were imaged using an Odyssey NIR imaging system (LI-COR Odyssey FC), at 700nm (GAPDH) and 800nm (protein of interest).

**
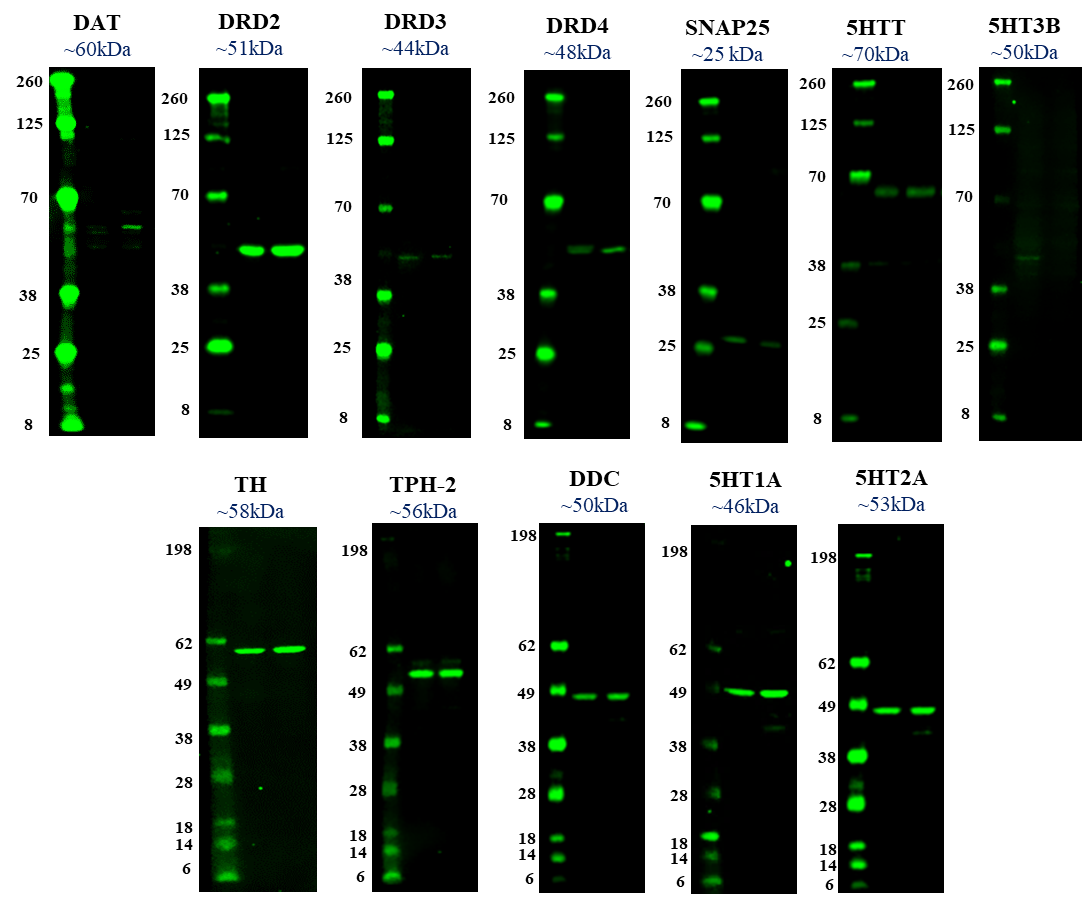
**

- 1. **Supplementary Figure 2**. Western blots showing the specificity of the antibodies.

Chameleon Pre-stained Protein Ladder (Includes 6 protein bands from 8 to 260 kDa); SeeBlue Ladder (Includes 8 protein bands from 6 to 198 kDa).

- 1. **Supplementary Figure 3.** Effect of dopaminergic neurons in VTA and SN on dopaminergic fibres in sgACC.

**(Top)** Correlations between DAT+ve fibre density in sgACC and dopaminergic neurons in VTA, Controls *rₛ*=0.277, *p*=0.338; DLB *rₛ*=-0.104, *p*=0.642; DLB+D *rₛ*=0.136, *p*=0.689; DLB-D s *rₛ*=-0.193, *p*=0.527; **(Bottom)** and dopaminergic neurons in SN within controls, DLB cases with and without depression, Controls *rₛ*=0.082, *p*=0.771; DLB *rₛ*=0.071, *p*=0.741; DLB+D *rₛ*=0.272, *p*=0.419; DLB-D s *rₛ*=-0.228, *p*=0.433.

**3.4 Supplementary Figure 4.** **Effect of dopaminergic neurons in VTA and SN on dopaminergic synapses in sgACC.**

**(Top)** Correlations between DAT+ve synapses in sgACC and dopaminergic neurons in VTA;

Controls *rₛ*=0.231, *p*=0.389; DLB *rₛ*=0.246, *p*=0.269; DLB+D *rₛ*=0.365, *p*=0.114; DLB-D *rₛ*=0.079, *p*=0.740. **(Bottom)** and DAT+ve synapses and dopaminergic neurons in SN, Controls *rₛ*=0.197, *p*=0.464; DLB *rₛ*=-0.074, *p*=0.659; DLB+D *rₛ*=-0.125, *p*=0.622; DLB-D *rₛ*=0.101, *p*=0.673.

**3.5 Supplementary Figure 5.** **Effect of dopaminergic neurons in VTA and SN on α-synuclein positive dopaminergic synapses in sgACC.**

**Top)** Correlations between α-synuclein positive DAT+ve synapses in sgACC and dopaminergic neurons in VTA; Controls *rₛ*=0.275, *p*=0.510; DLB *rₛ*=0.372, *p*=0.106; DLB+D *rₛ*=0.732, *p*=0.016*****; DLB-D *rₛ*=0.125, *p*=0.730. **Bottom)** and dopaminergic neurons in SN, Controls *rₛ*=0.067, *p*=0.876; DLB *rₛ*=-0.030, *p*=0.903; DLB+D *rₛ*=-0.070, *p*=0.857; DLB-D *rₛ*=0.258, *p*=0.471.

**References**

1. Braak H, Alafuzoff I, Arzberger T, Kretzschmar H, Del Tredici K. Staging of Alzheimer disease-associated neurofibrillary pathology using paraffin sections and immunocytochemistry. *Acta Neuropathologica* 2006; **112**(4)**:** 389-404.

2. McKeith IG, Dickson DW, Lowe J, Emre M, O'Brien JT, Feldman H *et al.* Diagnosis and management of dementia with Lewy bodies: third report of the DLB Consortium. *Neurology* 2005; **65**(12)**:** 1863-1872.

3. Thal DR, Rub U, Orantes M, Braak H. Phases of A beta-deposition in the human brain and its relevance for the development of AD. *Neurology* 2002; **58**(12)**:** 1791-1800.

4. Montine TJ, Phelps CH, Beach TG, Bigio EH, Cairns NJ, Dickson DW *et al.* National Institute on Aging-Alzheimer's Association guidelines for the neuropathologic assessment of Alzheimer's disease: a practical approach. *Acta Neuropathologica* 2012; **123**(1)**:** 1-11.

5. Walker L, McAleese KE, Thomas AJ, Johnson M, Martin-Ruiz C, Parker C *et al.* Neuropathologically mixed Alzheimer's and Lewy body disease: burden of pathological protein aggregates differs between clinical phenotypes. *Acta Neuropathol* 2015; **129**(5)**:** 729-748.

6. Monoranu CM, Apfelbacher M, Grünblatt E, Puppe B, Alafuzoff I, Ferrer I *et al.* pH measurement as quality control on human brain tissue: a study of the BrainNet Europe consortium. *Neuropath Appl Neuro* 2009; **35**(3)**:** 329-337.

7. Alexopoulos GS, Abrams RC, Young RC, Shamoian CA. Cornell Scale for Depression in Dementia. *Biol Psychiat* 1988; **23**(3)**:** 271-284.

8. Korner A, Lauritzen L, Abelskov K, Gulmann N, Brodersen AM, Wedervang-Jensen T *et al.* The geriatric depression scale and the Cornell Scale for Depression in Dementia. A validity study. *Nord J Psychiat* 2006; **60**(5)**:** 360-364.

9. Bradford MM. A rapid and sensitive method for the quantitation of microgram quantities of protein utilizing the principle of protein-dye binding. *Anal Biochem* 1976; **72:** 248-254.

10. Culvenor JG, McLean CA, Cutt S, Campbell BC, Maher F, Jakala P *et al.* Non-Abeta component of Alzheimer's disease amyloid (NAC) revisited. NAC and alpha-synuclein are not associated with Abeta amyloid. *Am J Pathol* 1999; **155**(4)**:** 1173-1181.
